# *In vitro* potential anthelmintic activity of *Biophytum petersianum* on *Haemonchus contortus*

**DOI:** 10.14202/vetworld.2018.1-4

**Published:** 2018-01-11

**Authors:** Priyo Sambodo, Joko Prastowo, Kurniasih Kurniasih, Sudarmanto Indarjulianto

**Affiliations:** 1 Doctoral program Faculty of Veterinary Medicine, Department of Parasitology, Universitas Gadjah Mada, Bulaksmur, Caturtunggal, Depok, Sleman, Daerah Istimewa Yogyakarta, 55281, Indonesia; 2 Department of Animal Science Papua University, Jalan Gunung Salju, Amban, Manokwari Papua Barat, 98314, Indonesia; 3 Department of Parasitology Universitas Gadjah Mada, Bulaksumur, Caturtunggal, Depok, Sleman, Daerah Istimewa Yogyakarta 55281, Indonesia; 4 Department of Pathology, Universitas Gadjah Mada, Bulaksumur, Caturtunggal, Depok, Sleman, Daerah Istimewa Yogyakarta 55281, Indonesia; 5 Department of Internal Medicine, Universitas Gadjah Mada, Bulaksumur, Caturtunggal, Depok, Sleman, Daerah Istimewa Yogyakarta 55281, Indonesia

**Keywords:** *Biophytum petersianum*, crude aqueous extract, *Haemonchus contortus*, scanning electron microscopy, sodium dodecyl sulfate polyacrylamide gel electrophoresis

## Abstract

**Aim:**

*Haemonchus contortus* is a major problem in small ruminants in Indonesia. The frequent use of the anthelmintic drugs has given rise to drug-resistant populations which increase the need for new anthelminthic compounds, particularly from endemic plants. This study evaluated the *in vitro* effects of *Biophytum petersianum* crude aqueous extract (BAE) as an anthelmintic compound against *H. contortus* adult worm isolated from goats.

**Materials and Methods:**

Adult worm collected from naturally infected abomasums were obtained from slaughtered goats on the day of slaughter. BAE was prepared in six different concentrations (10, 20, 40, 60, 80, and 100 mg/ml) which were tested for their efficacies on ten actively moving worms. Ivermectin (1 mg/ml) was included as a reference drug, while saline water was included as a control. The dead worms from anthelmintic test then went through sodium dodecyl sulfate polyacrylamide gel electrophoresis (SDS-PAGE) and scanning electron microscopy (SEM).

**Results:**

Highest mean mortality in treatments group both at 2 h and 4 h observations was BAE 10%. The SDS-PAGE analysis revealed the presence of five protein bands with molecular weights 9.3, 17.1, 50.0, 63.2, and 72.7 kDa based on BAE 10%. The SEM changes observed in the *in vitro* trials revealed the occurrence of interactions between the BAE and the cuticle.

**Conclusion:**

The SEM and SDS-PAGE analysis revealed ultrastructural structural changes and the decrease numbers of polypeptides on treated worms when compared to the control worms. It can thus be concluded that the BAE exhibits good anthelmintic activity against *H. contortus* adult worm.

## Introduction

*Haemonchus contortus* is a nematode class type of gastrointestinal parasite that causes haemonchosis. *H. contortus* is large and most pathogenic worms are found in the abomasums of goats and sheeps [1]. Economic losses due to haemonchosis in subtropic and tropic areas are usually caused by mortality, loss of production, retarded growth, and poor weight gain [2,3]. The previous studies from two provinces in Indonesia show that the prevalence of haemonchosis in goat is at 89.4% and an estimation of annual loss is around 1 million US dollar [4]. Gastrointestinal nematode infestation, especially *H. contortus*, is the major health problem for goats and sheeps in Indonesia [5].

Traditional medication is very frequently used in Papua, Indonesia, and people have been using these plants not only for the treatment of their own ailments but also for their domesticated animals. *Biophytum petersianum* (Oxalidaceae) commonly known as “rumput Kebar,” is a valuable medicinal plant in Papua Indonesia and the whole plant is utilized in the traditional system of medicine. Conventionally, *B. petersianum* has been used in mouthwashes, antidotes, and laxatives. Observation of the bovine fecal samples collected from Kebar district shows that the prevalence of the gastrointestinal parasite from 33 samples is at 0%.

The present study rationalizes the use of *B. petersianum* as anthelmintics against *H. contortus* adhering to standard parasitological procedures.

## Materials and Methods

### Ethical approval

All proceedings were approved by the Ethical Committee of Universitas Gadjah Mada (number 00116/04/LPPT/IX/2017).

### Collection and preparation of plant material

Plant materials were obtained from Manokwari district (133°03”25.8’ longitude; 00°48”31.1’ latitude, with a mean altitude of 110 m) West Papua province. Before being used, plant materials were dried in a ventilated room (drying room), cut into smaller pieces, and stored at 4°C until it is being used for extraction [6].

### Extraction

The plant materials (10 mg and 100 mg for each concentration) were soaked in 100 ml distilled water and incubated at 90°C for 15 min. The solution was filtered using filter paper, and the filtrate was stored in a freezer for 24 h [7].

### Parasite collection

Naturally infected abomasums were obtained from slaughtered goats on the day of slaughter from local slaughterhouses in Sleman districts, Yogyakarta. Abomasum was incised on the greater curvature and *H. contortus* was collected and placed in a Petri dish containing 0.62% water saline.

### Anthelmintic study

The anthelmintic study was conducted according to the method described by Alemu *et al*. [8] with slight modifications. *B. petersianum* crude aqueous extract (BAE) in different concentrations (10, 20, 40, 60, 80, and 100 mg/ml) was prepared for the anthelmintic assay. Ivermectin (1 mg/ml) was also included as a reference drug, while saline water was included as a control. Ten actively moving worms were placed in a 9 cm Petri dish containing 25 ml solution of BAE. Two replicates were performed for each treatment. Observations were made at 2 and 4 h after the treatment was done. The time of death of the worms was recorded after ascertaining that the worms neither moved when shaken rigorously nor when the body was poked with a needle. Five death worms were fixed in absolute ethanol and stored at 4°C until further use.

### Sodium dodecyl sulfate polyacrylamide gel electrophoresis (SDS-PAGE)

The dead worms from anthelmintic test were used for this stage. Briefly, the specimens were washed several times in phosphate-buffered saline (PBS) to remove all the debris and host-derived materials. The specimens were crushed in PBS 100 µl. The soluble proteins were isolated using centrifugation at 1200× *g* for 5 min and then underwent electrophoresis. SDS-PAGE was performed according to methods from Laemmli [9] with some minor modifications. The soluble protein samples as well as standard molecular weight markers (Thermo scientific, Lithuania) were boiled for 2 min in sample buffer in the ratio of 4:1 volume containing 50% glycerol, 10% SDS, 2.5% DTT and 0.05% (w/v) aqueous bromophenol blue as the marker dye. The electrophoresis was carried out at 100 V in a vertical slab gel system. After electrophoresis, the gels were stained in 0.2% (w/v) Coomassie blue.

### Scanning electron microscopy (SEM)

The dead worms from anthelmintic test were used in this stage. In brief, the fixed specimens were cleaned with cacodylate buffer for 2 h, pre-fixation in 2.5% glutaraldehyde for 48 h, fixation in 2% tannin acid which was washed 4 times with cacodylate buffer and distilled water, and then dehydrated in a series of ascending alcohols followed by drying in tertiary butanol. Dried specimens were coated with copper for 15 min and then observed with a SEM (JEOL JSM-5319LV, JEOL USA, Inc.).

### Statistical analysis

The data from anthelmintic test experiment were recorded and analyzed using SPSS 16.0 software. The results were expressed as a mean ± standard error of mean (SEM). The results from SEM and SDS-PAGE were descriptively analyzed.

## Results

### Anthelmintic study

The anthelmintic activity of crude aqueous extracts of *B. petersianum* at various concentrations is presented in Table-1. All concentrations showed a potential anthelmintic activity. Highest mean mortality was observed in treatment groups both at 2 h and 4 h at BAE 10%. The results of the ANOVA showed that BAE 10% and time of exposure were statistically significant towards *H. contortus* mortality (p=0.00).

**Table-1:** Mean efficacy±SD of *B. petersianum* extract on *H. contortus*.

Treatments	2 h	4 h
Ivermectin 1 mg/ml	10±0.00	10±0.00
BAE 1%	4±2.82	7±1.41
BAE 2%	2±1.41	6±1.41
BAE 4%	3±2.82	6.5±0.71
BAE 6%	1.5±0.71	6.5±3.54
BAE 8%	2±0.00	8±1.41
BAE 10%	6.5±3.53	9.5±0.71
Saline water	0±0.00	0±0.00

BAE=*Biophytum petersianum* crude aqueous extract; *H. contortus*=*Haemonchus contortus*; *B. petersianum*=*Biophytum petersianum*; SD=Standard deviation

### SDS-PAGE

**Figure-1:**
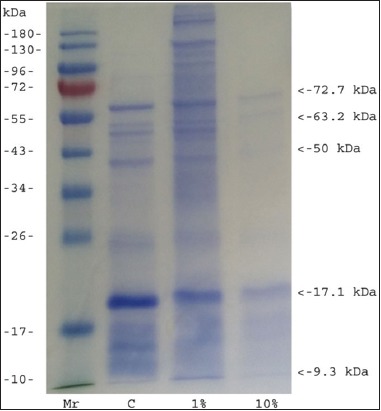
Sodium dodecyl sulfate polyacrylamide gel electrophoresis electropherogram of *Haemonchus contortus*. Lane 1 marker protein, lane 2, 3 and 4 parasite proteins.

Electrophoresis of soluble proteins of *H. contortus* post-treatment revealed the presence of five protein bands with molecular weights of 9.3, 17.1, 50.0, 63.2, and 72.7 kDa based on BAE 10%. The control group revealed the presence of nine prominent protein bands with molecular weights of 9.3, 11.8, 16.3, 24.8, 45.6, 52.4, 57.5, 60.3, and 69.3 kDa (Figure-1).

### SEM

**Figure-2:**
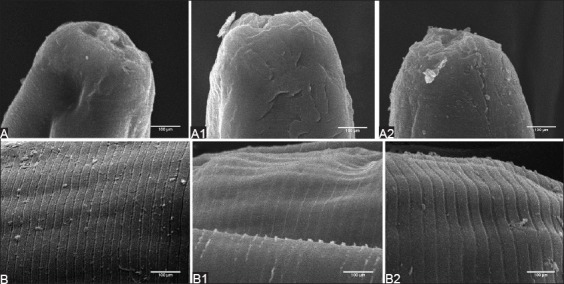
Scanning electron microscopic (SEM) image of the anterior end and the cuticle of the adult female *Haemonchus contortus*. (A and B) SEMs of the normal fresh worm; (A1 and B1) after incubation with 1 mg/ml BAE; (A2 and B2) after incubation with 10 mg/ml BAE.

The SEM micrographs showed changes in adult *H. contortus* after *in vitro* exposure to BAE 1% and 10% (Figure-2). The main changes that can be observed after 4 h of incubation with 1 mg/ml BAE are exfoliation of the tegument and a faded annuli transversal on the anterior end, revealing wrinkled and corrugated cuticular surface as well as a shallow annuli transversal (A1 and B1). Following 4 h of incubation with 10 mg/ml BAE, the main changes are exfoliation of the tegument and the loss of annuli transversal at the anterior end, showing a slightly more wrinkled cuticle as compared to normal with the discontinuation of annuli transversal (A2 and B2).

## Discussion

Preliminary phytochemical screening of *B. petersianum* has shown the presence of saponin, alkaloid, flavonoid, and tannin. Adult worms’ mortality in this study can be influenced by the presence of tannin in BAE. Tannins may react directly with adult worms by attaching to their “skin,” causing distress or indirectly by improving protein nutrition and increasing the immune system of the host [10]. The result of this study has similar result compare to Zaman *et al*. [11] who reported 100% mortality rate when treated with a herbal formulation based on aqueous extracts of leaves of *Azadirachta indica* and *Nicotiana tabacum*, flowers of *Calotropis procera* and seeds of *Trachyspermum ammi* against *H. contortus* adults worm. The result of this study also agrees with the findings of Eguale *et al*. [12] who reported that aqueous extract of *Coriandrum sativum* killed 45% of the tested *H. contortus*. Iqbal *et al*. [13] had also reported a positive effect of aqueous extracts in adult motility assay of *H. contortus* in their studies.

SDS-PAGE followed by Coomassie blue staining on BAE 10% showed ~5 polypeptides varying in size from <15 kDa to >70 kDa, while some studies have shown the greater numbers of polypeptides on untreated *H. contortus*. Anbu and Joshi [14] reported ~20 polypeptides varying in size from <15 kDa to >150 kDa and Jaiswal *et al*. [15] reported a total of 35 polypeptides of different molecular weights in female worms. The present result can be influenced by the presence of tannin in BAE. Tannin (in its condensed form) has an affinity for proline-rich proteins and the nematode cuticle is known to be a proline-rich structure that covers the body [16]. The proposed mechanisms of nematode control are that tannin disturbs the physiological processes of nematodes by binding to nematode proteins directly and subsequently blocking the parasites physiological processes and/or modifying host immune responses to eliminate infective larvae and adult worms [17].

The changes observed from the SEM in the *in vitro* trials revealed the occurrence of interactions between the BAE and the cuticle, which has an important role in many nematode functions, particularly in its motility and to selectively absorb nutrients. Cuticular changes with the presence of longitudinal and transverse wrinkles after *in vitro* exposure to walnut extract rich in condensed tannin on *Trichostrongylus colubriformis* adult worm were described by Hoste *et al*. [16]. The presence of wrinkles in the cuticle of *H. contortus* adult worm was also described by Martínez-Ortíz-De-Montellano *et al*. [18] and Yoshihara *et al*. [19]. Association of the structural cuticular changes described in this study points toward an inhibition of the motility of the parasite and the disturbance of the nematode’s nutrition, which might eventually lead to worm undernourishment.

## Conclusion

The SEM and SDS-PAGE analysis revealed ultrastructural structural changes and the decreased number of polypeptides on treated worms when compared to the control worms. It can thus be concluded that the BAE exhibits good anthelmintic activity against *H. contortus* adult worm.

## Authors’ Contributions

The study was arranged, designed, and supervised by KK. PS carried out sampling and laboratory analysis. JP, KK, and PS analyzed and interpreted the data while SI did the overall monitoring of the experiment and preparation of the manuscript. All authors have read and approved the final manuscript.
